# Acetato(1,10-phenanthroline-5,6-dione)silver(I) trihydrate

**DOI:** 10.1107/S1600536808000846

**Published:** 2008-01-25

**Authors:** Jonathan Onuegbu, Ray J. Butcher, Charles Hosten, Uche Charles Udeochu, Oladapo Bakare

**Affiliations:** a Department of Chemistry, Howard University, 525 College Street NW, Washington, DC 20059, USA

## Abstract

In the structure of the title compound, [Ag(C_2_H_3_O_2_)(C_12_H_6_N_2_O_2_)]·3H_2_O, the Ag^I^ atom is coordinated by both 1,10-phenanthroline-5,6-dione N atoms and one O atom from the acetate anion. The three water mol­ecules are involved in extensive hydrogen bonding to each other and to the acetate O and 1,10-phenanthroline-5,6-dione O atoms. In addition, there are weak C—H⋯O inter­actions.

## Related literature

For related literature, see: Allen (2002 ▶); Armaroli (2001 ▶); Burrows *et al.* (1995 ▶); Calderazzo *et al.* (1999 ▶, 2002 ▶); Calucci *et al.* (2006 ▶); Fox *et al.* (1991 ▶); Galet *et al.* (2005 ▶); Hilt *et al.* (1997 ▶); Lei *et al.* (1996 ▶); Leschke *et al.* (2002 ▶); Ma *et al.* (2002 ▶); Okamura *et al.* (2006 ▶); Onuegbu *et al.* (2007 ▶); Pallenberg *et al.* (1997 ▶); Paramonov *et al.* (2003 ▶); Paw & Eisenberg (1997 ▶); Ruiz *et al.* (1999 ▶); Scaltrito *et al.* (2000 ▶); Shavaleev *et al.* (2003*a* ▶, 2003*b* ▶); Titze *et al.* (1997 ▶); Uche *et al.* (2007 ▶); Whitesides *et al.* (1991 ▶).
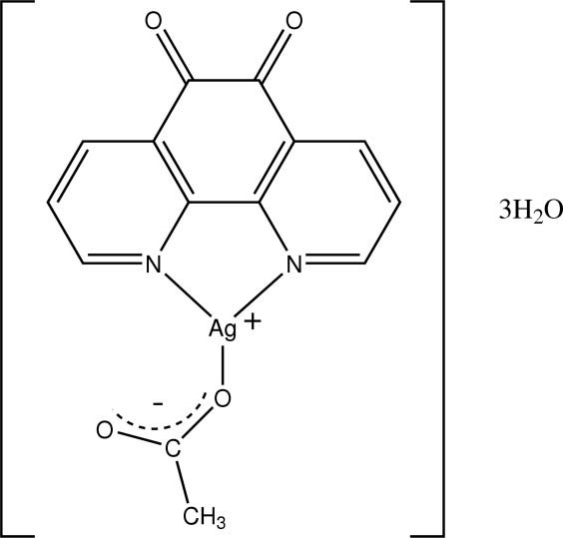


## Experimental

### 

#### Crystal data

[Ag(C_2_H_3_O_2_)(C_12_H_6_N_2_O_2_)]·3H_2_O
*M* *_r_* = 431.15
Triclinic, 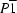

*a* = 6.6851 (11) Å
*b* = 9.6407 (17) Å
*c* = 12.818 (2) Å
α = 96.200 (2)°
β = 103.490 (2)°
γ = 104.629 (2)°
*V* = 765.0 (2) Å^3^
*Z* = 2
Mo *K*α radiation
μ = 1.36 mm^−1^
*T* = 103 (2) K
0.58 × 0.20 × 0.15 mm

#### Data collection

Bruker SMART 1000 CCD area-detector diffractometer
Absorption correction: multi-scan (*SADABS*; Sheldrick, 1996 ▶) *T*_min_ = 0.505, *T*_max_ = 0.821
8724 measured reflections
4315 independent reflections
4105 reflections with *I* > 2σ(*I*)
*R*_int_ = 0.020

#### Refinement

*R*[*F* ^2^ > 2σ(*F* ^2^)] = 0.028
*wR*(*F* ^2^) = 0.068
*S* = 1.07
4315 reflections
243 parameters
6 restraints
H atoms treated by a mixture of independent and constrained refinement
Δρ_max_ = 1.28 e Å^−3^
Δρ_min_ = −1.03 e Å^−3^

### 

Data collection: *SMART* (Bruker, 2003 ▶); cell refinement: *SAINT* (Bruker, 2003 ▶); data reduction: *SAINT*; program(s) used to solve structure: *SHELXS97* (Sheldrick, 2008 ▶); program(s) used to refine structure: *SHELXL97* (Sheldrick, 2008 ▶); molecular graphics: *SHELXTL* (Sheldrick, 2008 ▶); software used to prepare material for publication: *SHELXTL*.

## Supplementary Material

Crystal structure: contains datablocks global, I. DOI: 10.1107/S1600536808000846/ci2544sup1.cif

Structure factors: contains datablocks I. DOI: 10.1107/S1600536808000846/ci2544Isup2.hkl

Additional supplementary materials:  crystallographic information; 3D view; checkCIF report

## Figures and Tables

**Table:** 

Ag—O1*A*	2.1987 (14)
Ag—N2	2.2554 (17)
Ag—N1	2.3870 (18)

**Table:** 

O1*A*—Ag—N2	153.49 (6)
O1*A*—Ag—N1	133.64 (6)
N2—Ag—N1	71.57 (6)

**Table 2:** Hydrogen-bond geometry (Å, °)

*D*—H⋯*A*	*D*—H	H⋯*A*	*D*⋯*A*	*D*—H⋯*A*
O1*W*—H1*W*1⋯O2	0.80 (2)	2.01 (2)	2.773 (2)	158 (3)
O1*W*—H1*W*2⋯O1*W*^i^	0.81 (2)	2.05 (4)	2.781 (3)	151 (7)
O2*W*—H2*W*1⋯O1*W*^ii^	0.79 (2)	2.12 (2)	2.904 (2)	168 (4)
O2*W*—H2*W*2⋯O1*W*^iii^	0.81 (2)	2.00 (2)	2.805 (3)	170 (5)
O3*W*—H3*W*1⋯O2*A*	0.82 (2)	1.97 (2)	2.791 (2)	178 (4)
O3*W*—H3*W*2⋯O2*W*	0.82 (2)	1.95 (2)	2.769 (3)	172 (6)
C3—H3*A*⋯O1*A*^iv^	0.95	2.52	3.286 (3)	138
C8—H8*A*⋯O3*W*^v^	0.95	2.55	3.393 (3)	148
C10—H10*A*⋯O1^vi^	0.95	2.48	3.431 (3)	174

Symmetry codes: (i) 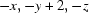; (ii) 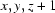; (iii) 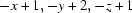; (iv) 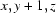; (v) 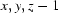; (vi) 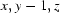.

**Fig. 1.**
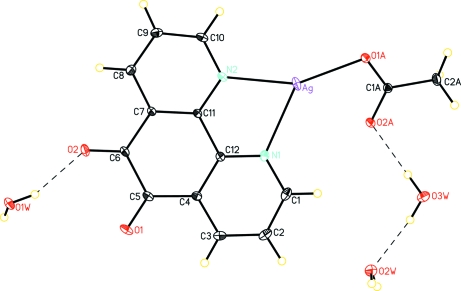
View of the complex, [AgL(CH3CO2)].3H2O, showing the atom-labelling scheme. Hydrogen bonds are indicated by dashed lines. Displacement ellipsoids are drawn at the 20% probability level.

**Fig. 2.**
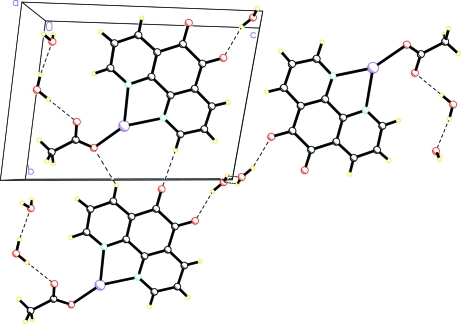
The molecular packing of the title compound, viewed approximately along the a axis. Dotted lines indicate hydrogen bonding interactions.

## supplementary crystallographic information

### Comment

The synthesis and understanding of multi-functional materials generated from spontaneously assembled molecules joined together non-covalently remain an important challenge in the science of molecules (Whitesides *et al.*, 1991; Burrows *et al.*, 1995; Galet *et al.*, 2005). Metal complexes bearing electro-active ligands with two or more accessible oxidation states have been synthesized and shown to exhibit unique electronic structures resulting from the combination of the oxidation states of the metal and ligands (Ma *et al.*, 2002; Calderazzo *et al.*, 1999; Calderazzo *et al.*, 2002; Calucci *et al.*, 2006; Galet *et al.*, 2005; Lei *et al.*, 1996; Okamura *et al.*, 2006).

While phendione usually binds to metals through it's imine N atoms (Onuegbu *et al.*, 2007), in some cases both the N and O donors are used simultaneously (Calderazzo *et al.*, 1999; Fox *et al.*, 1991; Shavaleev *et al.*, 2003*a*; Shavaleev *et al.*, 2003*b*; Ruiz *et al.*, 1999; Paw & Eisenberg, 1997). In light of these results, we have examined the coordination behavior of phendione with silver (Onuegbu *et al.*, 2007). In this paper we report the synthesis and characterization of the title compound, [Ag*L*(CH_3_CO_2_)].3H_2_O.

The structure of the title compound, shown in Fig. 1, is made up of a [Ag*L*(CH_3_CO_2_)] moiety and three water molecules. The Ag^I^ atom is coordinated to the two nitrogen atoms of a phendione ligand and one O from the acetate anion. There are no previous examples where a Ag^I^ is bound to only one phendione ligand. The C?O bond lengths in the phendione ligands (1.210 (3) and 1.213 (3) Å) are comparable to those values found in other such complexes (Allen, 2002). The metrical parameters for the phendione ligand is in the normal ranges observed for complexes where only the N atoms are coordinated to a metal (Allen, 2002). The Ag—N bond lengths (2.256 (2) and 2.387 (2) Å) are similar to those found in related phenanthroline and phendione derivatives of silver (Leschke *et al.*, 2002; Paramonov *et al.*, 2003; Pallenberg *et al.*, 1997; Titze *et al.*, 1997; Onuegbu *et al.*, 2007). In the title compound, silver is in a trigonal planar environment (Table 1).

In addition to the strong O—H···O hydrogen bonds (Table 2) formed by the water molecules to both the acetate and phendione O atoms, there are weak C—H···O hydrogen bonds between the hydrogen atoms on C2, C4 and C7 and either nitrate or phendione O atoms from an adjoining moiety.

### Experimental

A flask containing 1,10-phenanthroline hydrate (1.00 g, 5.04 mmol) and potassium bromide (5.95 g, 50.0 mmol) was placed in an ice bath. Concentrated sulfuric acid (20 ml) was added in small portions, followed by drop wise addition of concentrated nitric acid (10 ml). The resulting solution was heated for 2 h at 353–358 K and cooled to room temperature. The solution was then poured into 400 ml of water and neutralized with sodium bicarbonate, after which the phendione was extracted with dichloromethane, and recrystallized using a methanol-water mixture.

The title compound was synthesized in an atmosphere saturated with N_2_. To a solution of AgCH_3_CO_2_ (silver ethanoate) in 20 ml of 1:1 solution of methanol and water was added drop-wise a solution (20 ml) of 1:1 methanol: water mixture containing 0.05 g of phendione. The final yellowish solution was filtered and allowed to slowly evaporate for about three weeks yielding colorless needle-shaped crystals of the title compound suitable for X-ray studies.

### Refinement

C-bound H atoms were placed in geometrically idealized positions and constrained to ride on their parent atoms, with C—H = 0.95–0.98 Å and *U*_iso_(H) = 1.2*U*_eq_(C). The water H atoms were located as the highest peaks in the difference map after all other atoms had been located and refined. These all had reasonable O—H distanes and H—O—H angles and all formed hydrogen bonds with nearby O atoms. However for one H (H1W2) there was a close H1W2···H1W2 intermolecular contact (1.41 Å). When this atom was omitted from the refinement, the highest peak in the resulting difference map was in the same location. There were no other peaks which gave reasonable geometry. The water O—H distances were constrained to 0.82 Å.

### Crystal data

**Table:**

[Ag(C_2_H_3_O_2_)(C_12_H_6_N_2_O_2_)]·3H_2_O	*Z* = 2
*M**_r_* = 431.15	*F*_000_ = 432
Triclinic, *P*1	*D*_x_ = 1.872 Mg m^−^^3^
Hall symbol: -P 1	Mo *K*α radiation λ = 0.71073 Å
*a* = 6.6851 (11) Å	Cell parameters from 6131 reflections
*b* = 9.6407 (17) Å	θ = 2.0–30.6º
*c* = 12.818 (2) Å	µ = 1.36 mm^−^^1^
α = 96.200 (2)º	*T* = 103 (2) K
β = 103.490 (2)º	Needle, colorless
γ = 104.629 (2)º	0.58 × 0.20 × 0.15 mm
*V* = 765.0 (2) Å^3^	

### Data collection

**Table:**

Bruker SMART 1000 CCD area-detector diffractometer	4315 independent reflections
Radiation source: fine-focus sealed tube	4105 reflections with *I* > 2σ(*I*)
Monochromator: graphite	*R*_int_ = 0.020
*T* = 103(2) K	θ_max_ = 30.7º
φ and ω scans	θ_min_ = 2.5º
Absorption correction: multi-scan(SADABS; Sheldrick, 1996)	*h* = −9→9
*T*_min_ = 0.505, *T*_max_ = 0.821	*k* = −13→12
8724 measured reflections	*l* = −18→18

### Refinement

**Table:**

Refinement on *F*^2^	Secondary atom site location: difference Fourier map
Least-squares matrix: full	Hydrogen site location: inferred from neighbouring sites
*R*[*F*^2^ > 2σ(*F*^2^)] = 0.028	H atoms treated by a mixture of independent and constrained refinement
*wR*(*F*^2^) = 0.068	*w* = 1/[σ^2^(*F*_o_^2^) + (0.0247*P*)^2^ + 0.7651*P*] where *P* = (*F*_o_^2^ + 2*F*_c_^2^)/3
*S* = 1.07	(Δ/σ)_max_ = 0.007
4315 reflections	Δρ_max_ = 1.28 e Å^−^^3^
243 parameters	Δρ_min_ = −1.03 e Å^−^^3^
6 restraints	Extinction correction: SHELXL97 (Sheldrick, 1997), Fc^*^=kFc[1+0.001xFc^2^λ^3^/sin(2θ)]^-1/4^
Primary atom site location: structure-invariant direct methods	Extinction coefficient: 0.026 (2)

### Special details

**Table:**

Geometry. All e.s.d.'s (except the e.s.d. in the dihedral angle between two l.s. planes) are estimated using the full covariance matrix. The cell e.s.d.'s are taken into account individually in the estimation of e.s.d.'s in distances, angles and torsion angles; correlations between e.s.d.'s in cell parameters are only used when they are defined by crystal symmetry. An approximate (isotropic) treatment of cell e.s.d.'s is used for estimating e.s.d.'s involving l.s. planes.
Refinement. Refinement of *F*^2^ against ALL reflections. The weighted *R*-factor *wR* and goodness of fit *S* are based on *F*^2^, conventional *R*-factors *R* are based on *F*, with *F* set to zero for negative *F*^2^. The threshold expression of *F*^2^ > σ(*F*^2^) is used only for calculating *R*-factors(gt) *etc*. and is not relevant to the choice of reflections for refinement. *R*-factors based on *F*^2^ are statistically about twice as large as those based on *F*, and *R*- factors based on ALL data will be even larger.

### Fractional atomic coordinates and isotropic or equivalent isotropic displacement parameters (Å^2^)

**Table:**

	*x*	*y*	*z*	*U*_iso_*/*U*_eq_	
Ag	0.26396 (2)	0.328765 (18)	0.530264 (13)	0.02701 (7)	
O1	0.2255 (3)	0.94093 (16)	0.31707 (14)	0.0314 (3)	
O2	0.1697 (3)	0.73936 (18)	0.13835 (13)	0.0291 (3)	
O1A	0.3458 (2)	0.19712 (16)	0.65468 (11)	0.0237 (3)	
O2A	0.2011 (2)	0.34851 (16)	0.73262 (11)	0.0226 (3)	
N1	0.2608 (3)	0.57669 (19)	0.53824 (13)	0.0212 (3)	
N2	0.2125 (3)	0.37873 (18)	0.35977 (13)	0.0196 (3)	
C1	0.2860 (3)	0.6716 (3)	0.62821 (16)	0.0271 (4)	
H1A	0.2993	0.6381	0.6958	0.033*	
C2	0.2936 (4)	0.8165 (3)	0.62682 (18)	0.0304 (5)	
H2A	0.3128	0.8809	0.6923	0.036*	
C3	0.2725 (3)	0.8656 (2)	0.52843 (18)	0.0267 (4)	
H3A	0.2767	0.9642	0.5251	0.032*	
C4	0.2451 (3)	0.7677 (2)	0.43425 (15)	0.0193 (3)	
C5	0.2224 (3)	0.8171 (2)	0.32790 (17)	0.0212 (4)	
C6	0.1923 (3)	0.7039 (2)	0.22726 (16)	0.0196 (3)	
C7	0.1930 (3)	0.5547 (2)	0.24261 (14)	0.0168 (3)	
C8	0.1725 (3)	0.4507 (2)	0.15281 (16)	0.0229 (4)	
H8A	0.1591	0.4755	0.0823	0.028*	
C9	0.1722 (3)	0.3120 (2)	0.16871 (17)	0.0265 (4)	
H9A	0.1593	0.2394	0.1093	0.032*	
C10	0.1909 (3)	0.2797 (2)	0.27241 (17)	0.0242 (4)	
H10A	0.1884	0.1831	0.2824	0.029*	
C11	0.2144 (3)	0.51492 (19)	0.34543 (14)	0.0154 (3)	
C12	0.2408 (3)	0.6235 (2)	0.44269 (14)	0.0166 (3)	
C1A	0.2816 (3)	0.2439 (2)	0.73360 (15)	0.0187 (3)	
C2A	0.3012 (4)	0.1690 (3)	0.83090 (17)	0.0290 (4)	
H2AA	0.1579	0.1141	0.8334	0.043*	
H2AB	0.3701	0.2419	0.8976	0.043*	
H2AC	0.3883	0.1020	0.8251	0.043*	
O1W	0.2028 (3)	0.98250 (18)	0.03879 (14)	0.0279 (3)	
H1W1	0.190 (5)	0.927 (3)	0.081 (2)	0.045 (9)*	
H1W2	0.111 (9)	1.023 (8)	0.026 (7)	0.18 (3)*	
O2W	0.3820 (3)	0.8231 (2)	0.89715 (14)	0.0336 (4)	
H2W1	0.315 (6)	0.858 (4)	0.931 (3)	0.072 (13)*	
H2W2	0.505 (4)	0.874 (5)	0.910 (4)	0.097 (17)*	
O3W	0.3387 (3)	0.5397 (2)	0.93172 (14)	0.0354 (4)	
H3W1	0.296 (6)	0.483 (3)	0.874 (2)	0.053 (10)*	
H3W2	0.359 (9)	0.627 (3)	0.927 (5)	0.12 (2)*	

### Atomic displacement parameters (Å^2^)

**Table:**

	*U*^11^	*U*^22^	*U*^33^	*U*^12^	*U*^13^	*U*^23^
Ag	0.02657 (10)	0.03085 (11)	0.02929 (10)	0.01137 (7)	0.00869 (6)	0.01938 (7)
O1	0.0345 (8)	0.0148 (7)	0.0441 (9)	0.0076 (6)	0.0070 (7)	0.0094 (6)
O2	0.0393 (8)	0.0280 (8)	0.0268 (7)	0.0127 (7)	0.0137 (6)	0.0158 (6)
O1A	0.0339 (8)	0.0221 (7)	0.0216 (6)	0.0145 (6)	0.0113 (6)	0.0081 (5)
O2A	0.0278 (7)	0.0211 (7)	0.0230 (6)	0.0120 (6)	0.0078 (5)	0.0071 (5)
N1	0.0188 (7)	0.0254 (8)	0.0194 (7)	0.0053 (6)	0.0055 (6)	0.0056 (6)
N2	0.0197 (7)	0.0153 (7)	0.0238 (7)	0.0055 (6)	0.0047 (6)	0.0061 (6)
C1	0.0220 (9)	0.0387 (12)	0.0190 (8)	0.0060 (8)	0.0065 (7)	0.0020 (8)
C2	0.0280 (10)	0.0340 (12)	0.0245 (9)	0.0050 (9)	0.0077 (8)	−0.0069 (8)
C3	0.0261 (10)	0.0194 (9)	0.0318 (10)	0.0047 (8)	0.0076 (8)	−0.0026 (8)
C4	0.0180 (8)	0.0162 (8)	0.0225 (8)	0.0039 (6)	0.0048 (6)	0.0026 (7)
C5	0.0192 (8)	0.0151 (8)	0.0290 (9)	0.0043 (7)	0.0058 (7)	0.0061 (7)
C6	0.0201 (8)	0.0170 (8)	0.0247 (8)	0.0061 (7)	0.0083 (7)	0.0094 (7)
C7	0.0178 (8)	0.0149 (8)	0.0187 (8)	0.0049 (6)	0.0054 (6)	0.0050 (6)
C8	0.0242 (9)	0.0249 (10)	0.0199 (8)	0.0077 (7)	0.0062 (7)	0.0027 (7)
C9	0.0294 (10)	0.0214 (9)	0.0266 (9)	0.0079 (8)	0.0058 (8)	−0.0025 (7)
C10	0.0252 (9)	0.0143 (8)	0.0323 (10)	0.0064 (7)	0.0054 (8)	0.0035 (7)
C11	0.0141 (7)	0.0138 (8)	0.0181 (7)	0.0036 (6)	0.0041 (6)	0.0044 (6)
C12	0.0127 (7)	0.0171 (8)	0.0191 (8)	0.0030 (6)	0.0038 (6)	0.0041 (6)
C1A	0.0190 (8)	0.0174 (8)	0.0189 (8)	0.0038 (6)	0.0043 (6)	0.0044 (6)
C2A	0.0376 (11)	0.0334 (11)	0.0232 (9)	0.0173 (9)	0.0103 (8)	0.0142 (8)
O1W	0.0313 (8)	0.0228 (7)	0.0339 (8)	0.0100 (6)	0.0103 (6)	0.0146 (6)
O2W	0.0390 (9)	0.0294 (9)	0.0304 (8)	0.0073 (7)	0.0084 (7)	0.0045 (7)
O3W	0.0431 (10)	0.0308 (9)	0.0279 (8)	0.0114 (8)	0.0049 (7)	−0.0039 (7)

### Geometric parameters (Å, °)

**Table:**

Ag—O1A	2.1987 (14)	C6—C7	1.474 (3)
Ag—N2	2.2554 (17)	C7—C11	1.398 (2)
Ag—N1	2.3870 (18)	C7—C8	1.399 (3)
O1—C5	1.212 (2)	C8—C9	1.373 (3)
O2—C6	1.212 (2)	C8—H8A	0.95
O1A—C1A	1.270 (2)	C9—C10	1.383 (3)
O2A—C1A	1.257 (2)	C9—H9A	0.95
N1—C12	1.340 (2)	C10—H10A	0.95
N1—C1	1.341 (3)	C11—C12	1.484 (2)
N2—C11	1.343 (2)	C1A—C2A	1.504 (3)
N2—C10	1.346 (3)	C2A—H2AA	0.98
C1—C2	1.387 (3)	C2A—H2AB	0.98
C1—H1A	0.95	C2A—H2AC	0.98
C2—C3	1.384 (3)	O1W—H1W1	0.80 (2)
C2—H2A	0.95	O1W—H1W2	0.81 (2)
C3—C4	1.395 (3)	O2W—H2W1	0.79 (2)
C3—H3A	0.95	O2W—H2W2	0.81 (2)
C4—C12	1.399 (3)	O3W—H3W1	0.82 (2)
C4—C5	1.479 (3)	O3W—H3W2	0.82 (2)
C5—C6	1.536 (3)		
			
O1A—Ag—N2	153.49 (6)	C11—C7—C6	121.26 (16)
O1A—Ag—N1	133.64 (6)	C8—C7—C6	119.55 (17)
N2—Ag—N1	71.57 (6)	C9—C8—C7	118.65 (18)
C1A—O1A—Ag	104.47 (12)	C9—C8—H8A	120.7
C12—N1—C1	118.63 (18)	C7—C8—H8A	120.7
C12—N1—Ag	114.93 (12)	C8—C9—C10	119.10 (18)
C1—N1—Ag	126.37 (15)	C8—C9—H9A	120.5
C11—N2—C10	118.50 (17)	C10—C9—H9A	120.5
C11—N2—Ag	118.68 (12)	N2—C10—C9	122.98 (19)
C10—N2—Ag	122.73 (13)	N2—C10—H10A	118.5
N1—C1—C2	122.9 (2)	C9—C10—H10A	118.5
N1—C1—H1A	118.6	N2—C11—C7	121.57 (16)
C2—C1—H1A	118.6	N2—C11—C12	117.94 (16)
C3—C2—C1	118.84 (19)	C7—C11—C12	120.49 (16)
C3—C2—H2A	120.6	N1—C12—C4	122.01 (17)
C1—C2—H2A	120.6	N1—C12—C11	116.75 (17)
C2—C3—C4	118.8 (2)	C4—C12—C11	121.23 (16)
C2—C3—H3A	120.6	O2A—C1A—O1A	122.59 (17)
C4—C3—H3A	120.6	O2A—C1A—C2A	119.33 (17)
C3—C4—C12	118.85 (18)	O1A—C1A—C2A	118.08 (17)
C3—C4—C5	120.01 (18)	C1A—C2A—H2AA	109.5
C12—C4—C5	121.14 (17)	C1A—C2A—H2AB	109.5
O1—C5—C4	123.23 (19)	H2AA—C2A—H2AB	109.5
O1—C5—C6	119.30 (18)	C1A—C2A—H2AC	109.5
C4—C5—C6	117.47 (16)	H2AA—C2A—H2AC	109.5
O2—C6—C7	122.05 (18)	H2AB—C2A—H2AC	109.5
O2—C6—C5	119.57 (18)	H1W1—O1W—H1W2	115 (6)
C7—C6—C5	118.39 (16)	H2W1—O2W—H2W2	112 (5)
C11—C7—C8	119.19 (17)	H3W1—O3W—H3W2	115 (5)
			
N2—Ag—O1A—C1A	−165.35 (13)	C11—C7—C8—C9	0.6 (3)
N1—Ag—O1A—C1A	35.72 (16)	C6—C7—C8—C9	−179.60 (18)
O1A—Ag—N1—C12	167.44 (11)	C7—C8—C9—C10	0.3 (3)
N2—Ag—N1—C12	−2.82 (12)	C11—N2—C10—C9	0.5 (3)
O1A—Ag—N1—C1	−9.46 (19)	Ag—N2—C10—C9	−176.01 (15)
N2—Ag—N1—C1	−179.72 (17)	C8—C9—C10—N2	−0.9 (3)
O1A—Ag—N2—C11	−161.23 (13)	C10—N2—C11—C7	0.5 (3)
N1—Ag—N2—C11	2.85 (13)	Ag—N2—C11—C7	177.12 (13)
O1A—Ag—N2—C10	15.3 (2)	C10—N2—C11—C12	−179.30 (16)
N1—Ag—N2—C10	179.36 (17)	Ag—N2—C11—C12	−2.6 (2)
C12—N1—C1—C2	−0.3 (3)	C8—C7—C11—N2	−1.0 (3)
Ag—N1—C1—C2	176.46 (15)	C6—C7—C11—N2	179.17 (16)
N1—C1—C2—C3	0.5 (3)	C8—C7—C11—C12	178.76 (16)
C1—C2—C3—C4	−0.2 (3)	C6—C7—C11—C12	−1.1 (3)
C2—C3—C4—C12	−0.3 (3)	C1—N1—C12—C4	−0.2 (3)
C2—C3—C4—C5	−179.94 (19)	Ag—N1—C12—C4	−177.32 (13)
C3—C4—C5—O1	−0.3 (3)	C1—N1—C12—C11	179.68 (16)
C12—C4—C5—O1	−179.91 (19)	Ag—N1—C12—C11	2.52 (19)
C3—C4—C5—C6	−179.92 (17)	C3—C4—C12—N1	0.5 (3)
C12—C4—C5—C6	0.4 (3)	C5—C4—C12—N1	−179.88 (17)
O1—C5—C6—O2	−0.8 (3)	C3—C4—C12—C11	−179.36 (17)
C4—C5—C6—O2	178.84 (18)	C5—C4—C12—C11	0.3 (3)
O1—C5—C6—C7	178.90 (18)	N2—C11—C12—N1	−0.1 (2)
C4—C5—C6—C7	−1.4 (2)	C7—C11—C12—N1	−179.85 (16)
O2—C6—C7—C11	−178.52 (18)	N2—C11—C12—C4	179.77 (16)
C5—C6—C7—C11	1.8 (3)	C7—C11—C12—C4	0.0 (3)
O2—C6—C7—C8	1.7 (3)	Ag—O1A—C1A—O2A	−3.1 (2)
C5—C6—C7—C8	−178.06 (17)	Ag—O1A—C1A—C2A	176.13 (15)

### Hydrogen-bond geometry (Å, °)

**Table:**

*D*—H···*A*	*D*—H	H···*A*	*D*···*A*	*D*—H···*A*
O1W—H1W1···O2	0.80 (2)	2.01 (2)	2.773 (2)	158 (3)
O1W—H1W2···O1W^i^	0.81 (2)	2.05 (4)	2.781 (3)	151 (7)
O2W—H2W1···O1W^ii^	0.79 (2)	2.12 (2)	2.904 (2)	168 (4)
O2W—H2W2···O1W^iii^	0.81 (2)	2.00 (2)	2.805 (3)	170 (5)
O3W—H3W1···O2A	0.82 (2)	1.97 (2)	2.791 (2)	178 (4)
O3W—H3W2···O2W	0.82 (2)	1.95 (2)	2.769 (3)	172 (6)
C3—H3A···O1A^iv^	0.95	2.52	3.286 (3)	138
C8—H8A···O3W^v^	0.95	2.55	3.393 (3)	148
C10—H10A···O1^vi^	0.95	2.48	3.431 (3)	174

Symmetry codes: (i) −*x*, −*y*+2, −*z*; (ii) *x*, *y*, *z*+1; (iii) −*x*+1, −*y*+2, −*z*+1; (iv) *x*, *y*+1, *z*; (v) *x*, *y*, *z*−1; (vi) *x*, *y*−1, *z*.
